# Lifestyle Behaviors in Metabolically Healthy and Unhealthy Overweight and Obese Women: A Preliminary Study

**DOI:** 10.1371/journal.pone.0138548

**Published:** 2015-09-18

**Authors:** Sarah M. Camhi, Scott E. Crouter, Laura L. Hayman, Aviva Must, Alice H. Lichtenstein

**Affiliations:** 1 Exercise and Health Sciences Department, University of Massachusetts Boston, Boston, Massachusetts, United States of America; 2 Department of Kinesiology, Recreation, and Sport Studies, The University of Tennessee, Knoxville, Tennessee, United States of America; 3 Office of Vice Provost for Research, University of Massachusetts Boston, Boston, Massachusetts, United States of America; 4 Department of Public Health and Community Medicine, Tufts University School of Medicine, Boston, Massachusetts, United States of America; 5 JM USDA Human Nutrition Research Center on Aging, Tufts University, Boston, Massachusetts, United States of America; University of Geneva, SWITZERLAND

## Abstract

**Background:**

Few studies have examined dietary data or objective measures of physical activity (PA) and sedentary behavior among metabolically healthy overweight/obese (MHO) and metabolically unhealthy overweight/obese (MUO). Thus, the purpose is to determine whether PA, sedentary behavior and/or diet differ between MHO and MUO in a sample of young women.

**Methods:**

Forty-six overweight/obese (BMI ≥25 kg/m^2^) African American and Caucasian women 19–35 years were classified by cardiometabolic risk factors, including elevated blood pressure, triglyceride, glucose and C-reactive protein, low high density lipoprotein, and insulin resistance (MUO ≥2; MHO, <2). Time (mins/day) in light, moderate, vigorous PA, and sedentary behavior were estimated using an accelerometer (≥3 days; ≥8 hrs wear time). Questionnaires were used to quantify sitting time, TV/computer use and usual daily activity. The Block Food Frequency Questionnaire assessed dietary food intake. Differences between MHO and MUO for lifestyle behaviors were tested with linear regression (continuous data) or logistic regression (categorical data) after adjusting for age, race, BMI, smoking and accelerometer wear and/or total kilocalories, as appropriate.

**Results:**

Women were 26.7±4.7 years, with a mean BMI of 31.1±3.7 kg/m^2^, and 61% were African American. Compared to MUO (n = 9), MHO (n = 37; 80%) spent less mins/day in sedentary behavior (difference: -58.1±25.5, p = 0.02), more mins/day in light PA (difference: 38.2±16.1, p = 0.02), and had higher daily METs (difference: 0.21±0.09, p = 0.03). MHO had higher fiber intakes (g/day of total fiber, soluble fiber, fruit/vegetable fiber, bean fiber) and daily servings of vegetables; but lower daily dairy servings, saturated fat, monounsaturated fat and *trans* fats (g/day) compared to MUO.

**Conclusion:**

Compared to MUO, MHO young women demonstrate healthier lifestyle habits with less sedentary behavior, more time in light PA, and healthier dietary quality for fat type and fiber. Future studies are needed to replicate findings with larger samples that include men and women of diverse race/ethnic groups.

## Introduction

Obesity is a complex and heterogeneous condition with considerable phenotypic variation. One recent subgroup of interest is the metabolically “healthy” obese (MHO). These individuals have more favorable lipid profiles [1], higher insulin sensitivity [2], and lower risks for cardiovascular disease [3] compared to their metabolically”unhealthy” obese (MUO) counterparts.

Lifestyle behaviors, such as physical activity (PA), diet and sedentary behaviors may differentiate MHO and MUO phenotypes, and suggest possible targets for intervention strategies to modify and lower cardiometabolic risk in individuals with excess body weight. Previous research has shown conflicting results concerning PA levels between MHO and MUO: some studies have reported significantly greater levels of moderate-to-vigorous PA (MVPA) [4–6] whereas others have reported no significant differences [1, 2, 7–10]. However, most research utilized subjective methods such as questionnaires to estimate PA. Objective measures of physical activity levels have been estimated by overall energy expenditure (kcals/day) between MHO and MUO, however, these studies have been performed in primarily sedentary postmenopausal women, limiting the generalizability of the data and results to other populations [1, 2, 9]. Other limitations in existing research which compare MHO and MUO are measures for various PA types or domains, such as flexibility or strength training, which have been associated with lower body weight and more favorable cardiometabolic risk factors [11, 12].

Few studies have examined sedentary behavior between MHO and MUO groups. No differences have been reported between MHO and MUO for television time, a subjective measure of one type of sedentary behavior [7, 13]; however, other types of sedentary behavior measures and/or assessments of sedentary time such as computer use, sitting time or objective assessment have not yet been evaluated.

Recent data suggest that higher overall dietary quality is associated with the MHO profile [10, 14], but other studies have not identified significant differences between MHO and MUO for specific food groups [7] or macronutrient or micronutrient intakes [8]. However, these data were reported from studies of middle aged adults [7], Koreans [8] and Irish adults [10], precluding generalizability to a young sample of African American and Caucasian women.

The aim of this study was to compare PA, sedentary behavior and diet between MHO and MUO in a group of young African American and Caucasian women. MHO phenotypes track from childhood to adulthood [15], and MHO is more likely to occur in African American women [6] making this age, gender and race/ethnicity group a critical subgroup to include. We hypothesized that MHO women would have more time in objectively measured physical activity, less time in sedentary behavior and demonstrate healthier dietary intake compared to MUO women.

## Methods

Women were recruited from the student, staff and faculty members at the University of Massachusetts, Boston, and also from the surrounding city and suburbs. Criteria for inclusion were African American and Caucasian females, U.S. born, 19–35 years of age and a body mass index (BMI) of 25–39.9 kg/m^2^. Women were excluded if they were pregnant (currently or within past 6 months), breastfeeding, reported a body weight change of ≥5kg (11 lbs) or major change in dietary or exercise habits in past 6 months, reported a diagnosis of cardiovascular disease, diabetes (type I or II), thyroid disease or HIV/AIDS, use of medications to treat elevated cholesterol, blood pressure or glucose, or taking any dietary supplements with known effects on cardiometabolic risk factors (ie., antioxidants, vitamin E, aspirin, fish oil). All methods and procedures were approved by the University of Massachusetts Boston Institutional Review Board, and all participants signed an informed consent prior to measurement.

Women self-reported demographic information, personal health history, family health history, age, race/ethnicity, and smoking status (never, former or current). All measures were taken by certified technicians. Height was measured using a stadiometer after removing shoes, rounding up to the nearest 0.1 centimeter. Weight was measured to the nearest 0.1 kilogram with a digital scale Seca (Chino, CA) after all outer clothing, heavy pocket items and shoes were removed. Both height and weight were taken twice, with a third measurement obtained if the first two measures were > 0.5 cm or 0.5 kg, apart. BMI was calculated by dividing weight (kg) by height squared (m^2^). Waist circumference (WC) was measured in duplicate at the level of the iliac crest [16], and hip circumference was measured in duplicate at the maximal circumference when viewed from the side. Waist-to-hip ratio (WHR) was calculated by dividing waist circumference by hip circumference. Means of the height, weight and waist circumference values were used in all analyses.

Blood pressure was determined using an aneroid sphygmomanometer (American Diagnostic Corporation, Hauppauge, NY) using the right arm after a 5 minute rest. Mid-upper-arm circumference was used to determine the proper cuff size for accurately measuring the participant’s blood pressure. A second measure was taken 1–2 minutes later, and the average systolic and diastolic measurements were used in all analyses.

All women fasted for a minimum of 10 hours prior to the venipuncture. After a 30 minute clotting period at room temperature, approximately 5 mL of blood were centrifuged at 3500 rpm for 15 minutes. Immediately following centrifugation, samples were aliquoted and frozen at -80°C until biochemical analyses. All biochemical assays were conducted by the Nutrition Evaluation Laboratory at the Jean Mayer USDA Human Nutrition Research Center on Aging at Tufts University (Boston, MA). Serum was analyzed for fasting glucose, total cholesterol, triglyceride, and high density lipoprotein cholesterol (HDL), low and very-low density lipoprotein cholesterol (LDL and VLDL), glucose, insulin, C-reactive protein (hs-CRP). Lipid and glucose concentrations were measured on an automated chemistry analyzer (Olympus AU400) using reagents and calibrator from Beckman-Coulter. Insulin concentrations were measured using a radioimmunoassay (Human Insulin Specific RIA Kit; Linco Research Inc, St Charles, MO). Serum hs-CRP was measured immunoturbidimetrically (DiaSorin, Inc., Stillwater, MN) using a Roche Cobas Fara centrifugal clinical chemistry analyzer (Roche Diagnostics, Indianapolis, IN). The homeostasis model of insulin resistance (HOMA-IR) was calculated as [(fasting glucose (mmol/L)*fasting insulin(μU/ml)]/22.5[17].

MHO was defined as presence of overweight or obesity (25 to 39.9 kg/m^2^) and ≤2 abnormal cardiometabolic risk factors including elevated blood pressure (≥130 or ≥85 mmHg), triglyceride ≥150 mg/dL and glucose (≥100 mg/dL); low HDL-C (<50 mg/dL); insulin resistance (HOMA-IR > 90^th^ percentile 5.49), and systemic inflammation (hs-CRP > 90^th^ percentile 14.4 mg/L) [6].

### Physical Activity and Sedentary Behavior Assessment

Participants wore an ActiGraph GT3X+ accelerometer (Pensacola, FL) for a minimum of 7 days around their waist fixed with an elastic belt during all waking hours, except during water activities. The accelerometer data was used to estimate time spent in sedentary (<100 counts per minute (cpm) [18], light (100–759 cpm), moderate (760–5998 cpm), and vigorous (≥5999 cpm) PA [19, 20], steps per day [21] and metabolic equivalents (METS) [22]. Wear time was set at a minimum of 8 hours per day on a minimum of 3 days, with non-wear time defined as 60 minutes of continuous zeroes with a 2 minute interruption [23]. MVPA bouts of activity were deduced into a minimum of 10 minutes with allowance for a 2 minute interruption with a minimum of 760 cpm.

The 7-Day Physical Activity Recall [24] was interviewer administered to participants after completion of accelerometer wear to estimate minutes per day and/or hours spent in moderate, hard and/or very hard PA, sleep, resistance training and flexibility training. Hours of sleep was calculated as the time between the participant reporting going to bed the previous day and waking up the next morning for the previous 7 days. Time spent in sleep (1 MET), light (1.5 METs), moderate (4 METs), hard (6 METs), and very hard (10 METs) activities for the past 7 days are multiplied by their respective MET values and then summed [25]. An estimate of total kilocalories of energy expenditure per day was calculated. This instrument has been shown to be reliable and valid in young women [26].

Women were also asked questions which estimated time spent watching TV/videos and computer use with “Over the past 30 days, outside of work and school (as applicable), on average, how many hours per day did you 1) sit and watch television or videos and 2) use a computer (or iPad/iPhone/smart phone) to play games, surf the internet, view social media, or message others.” [27] We also asked women to pick a category which best describes their “usual daily activity” such as employment, housework, going to/attending classes/school. Categories were 1) sitting during the day without much walking; 2) standing or walking during the day, but do not have to carry or lift things very often; 3) lifting light loads or climbing stairs or hills often; 4) heavy work or carrying heavy loads. [27] Finally, we asked about time spent sitting or reclining at work, home or school during a typical weekday and typical weekend day. Weekdays and weekend day were deduced to sitting time (mins/week) by averaging with the following equation: (weekday value * 5 and the weekend value *2)/7 [28].

### Dietary Assessment

The 2005 Block Food Frequency Questionnaire (FFQ) was used to estimate habitual dietary intake [29]. Participants self-reported consumption of food intake over the past year from a 110 item questionnaire and data was reduced into estimates food groups and macro- and micronutrients at NutritionQuest (Berkeley, CA).

### Statistical Methods

All analyses were tested with SAS, version 9.3 (Cary, NC) with a p-value of <0.05 considered significant. T-tests and chi-square tests were used to compare demographic characteristics between MHO and MUO. Mixed linear regression models compared mean values for MHO and MUO for continuous PA, sedentary behavior and diet. Logistic regression analyses were used to compare categorical variables between MHO and MUO (computer/TV time, usual daily activity). All linear and logistic PA and sedentary behavior analyses adjusted for age, race, BMI, smoking status and accelerometer wear time (as applicable for accelerometer data). All dietary linear analyses adjusted for age, race, BMI and/or total calories.

The total sample size of eligible overweight/obese African American and Caucasian women was n = 55. Women were not included in the analyses if they had less than 3 days of 8 hours of wear or incomplete or missing accelerometer data (n = 6), or missing cardiometabolic data (n = 3), resulting in a final sample size of n = 46.

## Results

Approximately 61% of the sample was African American, and 80% were classified as MHO. Average age was (mean ± SD) 26.7 ±4.7 years with an average BMI of 31.1 ± 3.7 kg/m^2^. On average, the accelerometer was worn 832.6 ± 103.5 minutes per day for 7.0 ± 2.0 days. There were no significant differences between the MHO and MUO groups for age, race distribution, smoking status, accelerometer wear time or valid days of accelerometer wear. Women who were classified as MUO had significantly higher weight, height, BMI, waist circumference, WHR, and VLDL, HDL, triglyceride, glucose and CRP concentrations compared with MHO. Women in MHO and MUO had similar levels of total cholesterol, low-density lipoprotein cholesterol, systolic and diastolic blood pressure, and HOMA-IR values. Demographic and cardiometabolic risk factors are presented in Table 1.

**Table 1:** Demographic and cardiometabolic characteristics of final analytic sample (n = 46) (mean ± SD).

	Total Sample	MHO	MUO
**Demographic Characteristics**			
n (% of total)	46; 100%	n = 37; 80%	n = 9; 20%
Age (yrs)	26.7 ± 4.7	26.6 ± 4.7	26.9 ± 5.0
Race/Ethnicity (n (%) AA)	28 (61)	24 (65)	4 (44)
Smoking Status n (%)			
Never	37(80)	29 (80)	8 (89)
Former	8 (18)	7 (19)	1 (11)
Current	1 (2)	1 (<1)	0 (0)
**Cardiometabolic Variables**			
Weight (kg)	84.4 ± 13.5	81.3 ± 12.8	97.3 ± 7.5*
Height (cm)	164.5 ± 7.2	163.2 ± 6.6	169.9 ± 7.5*
BMI (kg/m^2^)	31.1 ± 3.7	30.4 ± 3.4	33.9 ± 3.9*
Waist Circumference (cm)	98.5 ± 10.7	95.2 ± 8.9	112.1 ± 5.7*
Waist Hip Ratio	0.88 ± 0.08	0.87 ± 0.08	0.96 ± 7.5*
Total Cholesterol (mg/dL) (mmol/L)	174.4 ± 34.9 4.5 ± 0.9	174.1 ± 34.8 4.5 ± 0.9	175.8 ± 37.1 4.5 ± 1.0
LDL Cholesterol (mg/dL) (mmol/L)	103.3 ± 33.4 2.7 ± 0.9	102.0 ± 33.1 2.6 ± 0.9	108.7 ± 36.1 2.8 ± 0.9
VLDL Cholesterol (mg/dL) (mmol/L)	16.0 ± 7.7 0.4 ± 0.2	13.8 ± 5.1 0.4 ± 0.1	24.9 ± 10.2* 0.6 ± 0.3
HDL Cholesterol (mg/dL) (mmol/L)	55.2 ± 13.8 1.4 ± 0.4	58.3 ± 13.2 1.5 ± 0.3	42.4 ± 6.6* 1.1 ± 0.2
Triglycerides (mg/dL)^ (mmol/L)	80.0 ± 38.1 0.9 ± 0.4	69.2 ± 25.3 0.8 ± 0.3	124.6 ± 50.2* 1.4 ± 0.6
Systolic BP (mmHg)	107.3 ± 9.3	107.0 ± 9.5	108.6 ± 9.1
Diastolic BP (mmHg)	70.1 ± 7.6	69.1 ± 6.7	74.2 ± 10.1
Glucose (mg/dL) (mmol/L)	91.0 ± 10.1 5.1 ± 0.6	89.2 ± 5.5 5.0 ± 0.3	98.0 ± 19.1* 5.4 ± 1.1
HOMA-IR	4.3 ± 10.0	2.5 ± 1.0	11.8 ± 21.9
hs-C-Reactive Protein (mg/L) (nmol/L)	5.4 ± 6.1 51.4 ± 58.1	4.5 ± 4.9 42.8 ± 46.7	9.1 ± 8.9* 86.7 ± 84.8

^*^ p < 0.05 for comparison of unadjusted means for MHO vs. MUO (t-tests for continuous variables; chi-square for categorical variables).

^ Non-normally distributed variables log transformed for t-test

MHO women had significantly higher levels of light PA compared to MUO (difference between MHO and MUO; 38. ± 16.1 mins/day, p = 0.02) when measured using accelerometry. MHO also had greater average daily METs (per minute) compared with MUO (difference: 0.21 ± 0.09 METS, p = 0.03) (Table 2). Other accelerometer measures, including moderate PA, vigorous PA, MVPA bouts, total activity counts, and steps per day, were similar between MHO and MUO women. Measures from the 7-day self-reported physical activity recall questionnaire did not show any significant differences between MUO and MHO for any intensity of activity, strength training or flexibility training (Table 2).

**Table 2:** Adjusted means and standard error* for PA variables between MHO (n = 37) and MUO (n = 9).

	MHO	MUO	Difference	p-value
**Accelerometer Derived Data**			** **	** **
Light PA (mins/day)	143.1 ± 13.7	104.8 ± 19.0	38.2 ± 16.1	**0.02**
Moderate PA (mins/day)	102.9 ± 11.9	84.9 ± 16.6	18.0 ± 14.1	0.21
Vigorous PA (mins/day)	1.8 ± 0.5	0.3 ± 0.9	1.5 ± 1.0	0.14
MVPA (mins/day)	103.8 ± 12.2	84.3 ± 17.0	19.50 ± 14.5	0.19
MVPA Bouts (#/day)	0.9 ± 0.4	1.3 ± 0.6	-0.4 ± 0.5	0.46
MVPA Bouts (mins/bout)	12.3 ± 1.3	10.9 ± 1.9	1.5 ± 1.6	0.36
Total Activity Counts (counts/day)	254,640 ± 35,942	203,547 ± 50,005	51,093 ± 42,449	0.24
METs^ (per minute)	1.84 ± 0.08	1.63 ± 0.11	0.21 ± 0.09	**0.03**
Steps (steps/day)	12,320 ± 1,337	10,144 ± 1,860	2,176 ± 1,579	0.18
**Questionnaire Derived Data**				
Light^+^ (hrs/week)	151.4 ± 2.6	153.3 ± 3.6	-1.9 ± 3.0	0.53
Moderate^+^ (hrs/week)	7.5 ± 2.5	6.0 ± 3.5	1.5 ± 2.9	0.61
Hard^+^ (hrs/week)	1.0 ± 0.7	0.6 ± 0.9	0.3 ± 0.8	0.66
Very Hard^+^ (hrs/week)	0.4 ± 0.4	0.2 ± 0.6	0.2 ± 0.5	0.66
Energy Expenditure (kcals/kg/week)	275.0 ± 7.9	267.9 ± 11.0	7.1 ± 9.0	0.44
Strength Training (mins/week)	23.4 ± 14.7	14.7 ± 20.7	8.7 ± 16.9	0.61
Flexibility Training (mins/week)	44.5 ± 7.0	38.8 ± 9.8	5.7 ± 8.0	0.48
**Usual Occupational Activity**				
Sitting n (%)	6 (16)	3 (33)		0.40
Standing or walking without carrying heavy loads n (%)	25 (68)	4 (45)		
Lifting light loads, climbing stairs often n (%)	6 (16)	2 (22)		
Heavy work or carrying heavy loads n (%)	0 (0)	0 (0)		

Key: PA: physical activity, MVPA: Moderate and vigorous physical activity.

**Bold** text indicates significant findings p<0.05.

*adjusted for BMI, age, race, smoking and wear time (for accelerometer derived data)

^ METs via Crouter et al., 2010 equation [22]

^+^ MET equivalents for 7 day Recall Questionnaire are light (1.5 METS), moderate (4 METS), hard (6 METS) and very hard (10 METS) [25]

MHO women spent significantly less time in sedentary behaviors, measured via accelerometry, than MUO (difference between MHO and MUO: -58.1 ± 25.5 mins/day p = 0.02) (Table 3). Questionnaire data for sitting time, TV time, computer use, or usual daily activity were similar for MHO and MUO (Table 3).

**Table 3:** Adjusted means*(A) and odds ratios (B) for sedentary behavior between MHO (n = 37) and MUO (n = 9).

A)	**MHO**	**MUO**	**Difference**	**p-value**
**Accelerometer Derived Data**				
Sedentary behavior (mins/day)	585.8 ± 21.6	643.8 ± 30.1	-58.1 ± 25.5	**0.02**
**Questionnaire Derived Data**				
Sitting Time (mins/day)	299.0 ± 56.4	422.4 ± 78.6	-123.4 ± 64.0	0.06
B)	Adjusted OR (95% CI)**	**p-value**		
TV (>3hrs/day)	0.2 (0.03–1.9)	0.18		
Computer (>3hrs/day)	0.7 (0.09–5.2)	0.70		
Usual Daily Activity^"^ (Sitting)	0.5 (0.07–3.4)	0.46		

*adjusted for BMI, age, race, smoking and wear time (accelerometer-derived data)

" Categories of usual daily activity were divided into 1) sitting vs. 2) standing, walking, and lifting loads

** MHO compared to MUO

**Bold** text indicates significant findings p<0.05.

MHO women reported higher fiber intakes than MUO women (difference between MHO and MUO: 6.3 ± 2.8 g, p = 0.03), soluble fiber intake (difference: 1.8 ± 0.7 g, p = 0.02), and lower intakes of saturated fat (difference: -4.0 ± 1.9 g, p = 0.04), monounsaturated fat (difference: -4.0 ± 1.9 g, p = 0.04), *trans* fat (difference: -0.8 ± 0.4 g, p = 0.03) (Table 4).

**Table 4:** Adjusted means* for macronutrient, micronutrient and summary diet variables between MHO (n = 37) and MUO (n = 9).

	MHO		MUO		p-value*
**Selected Macro/Micronutrients**	Mean	SE	Mean	SE	
Total kcals	1,850	204	1,900	414	0.92
Carbohydrates (% kcal)	49.1	1.3	47.8	2.6	0.66
Sugar (g)	109.4	7.4	112.5	15.1	0.86
Fiber (g)	21.3	1.2	14.9	2.4	**0.03**
Soluble Fiber (g)	6.6	0.3	4.8	0.6	**0.02**
Fat (% kcal)	35.6	0.9	37.5	1.9	0.39
Polyunsaturated Fat (g)	17.9	0.7	16.2	1.3	0.25
Monounsaturated Fat (g)	29.7	0.8	33.8	1.6	**0.04**
*Trans* Fat (g)	2.4	0.2	3.3	0.3	**0.03**
Saturated Fat (g)	23.3	0.8	27.3	1.6	**0.04**
Cholesterol (mg)	229.8	14.6	283.4	29.6	0.12
Protein (% kcal)	15.2	0.6	15.5	1.2	0.83
Sodium (mg)	3,198	85	3,002	173	0.33
Calcium (mg)	801	43	979	87	0.09
**Summary Dietary Variables**					
Total Fruit (daily servings of fruits and juices)	1.7	0.2	1.2	0.4	0.18
Total Vegetables (daily servings)	3.9	0.4	2.0	0.7	**0.03**
Total Grains (daily servings)	4.8	0.3	5.0	0.6	0.81
Whole Grains (daily servings)	0.7	0.1	0.6	0.2	0.62
Dairy (milk, yogurt and cheese) (daily servings)	1.0	0.1	1.7	0.3	**0.04**
Meat, Fish, Poultry, Beans, Eggs (daily servings)	2.4	0.1	2.3	0.3	0.88
Fats, oils, sweets and soda (daily servings)	3.2	0.3	3.9	0.5	0.26
Alcohol (% total kcal)	3.9	0.7	1.6	1.4	0.17
Fiber—Beans (g)	2.9	0.3	1.2	0.6	**0.03**
Fiber—Fruits and vegetables (g)	10.0	0.9	5.2	1.8	**0.03**
Fiber—Grains (g)	8.0	0.7	7.9	1.5	0.97
Sugar Sweetened Beverages (g)	262.4	68.1	342.7	138.2	0.62
Sweets & Desserts (%kcal)	12.8	1.3	17.0	2.6	0.17

*adjusted for BMI, age, race and kcals

**Bold** text indicates significant findings p<0.05.

MHO women, compared to MUO women, reported consuming higher daily servings for vegetables (difference: 1.9 ± 0.8 servings, p = 0.03), fiber from vegetables (difference: 4.7 ± 2.1 g, p = 0.03), and fiber from beans (difference: 1.7 ± 0.8 g, p = 0.03), and lower daily servings of dairy (difference: -0.7 ± 0.3 servings, p = 0.04) (Table 4). Further analysis of the dairy intake showed that servings of milk was significantly lower in MHO women compared to MUO women (MHO: 0.4 ± 0.1 vs. MUO: 1.0 ± 0.2 cups; p = 0.02) with no significant difference for yogurt or cheese intake (data not shown).

## Discussion

The current study demonstrates that MHO young African American and Caucasian women exhibit healthier lifestyle behaviors: MHO women spent less time per day in sedentary behavior and more time per day in light PA, resulting in an overall higher daily energy expenditure compared with MUO. MHO women also demonstrated healthier overall dietary intake with higher intake of vegetables, fiber, and lower intake of saturated fat and dairy products.

Key differences in physical activity and sedentary behavior were found using objective and rigorous assessment, and to our knowledge, this is the first objective assessment of sedentary behavior comparing MHO and MUO phentoypes. The accelerometry data show that time spent per day in sedentary behaviors was significantly lower in MHO women. MHO women in our study spent approximately 58 minutes less in sedentary behavior per day compared to MUO, and this caused displacement of both light PA (addition of approximately 38 minutes per day, p = 0.02) and MVPA (addition of approximately 20 minutes per day, p = 0.19). Light PA has been associated with lower glucose concentrations indicating effects of lower intensity activity on cardiometabolic health [30], and research in young adults has shown that simply replacing sitting with either standing or walking can improve insulin sensitivity [31]. Substituting 30 mins/day of sedentary time with equal amounts of light PA was associated with improvements in overall physical health in older adults [32]. Even substituting 10 minutes of sedentary time for MVPA has been reported to be positively associated with cardiometabolic risk factors also in older adults [33]. The overall lower levels of sedentary behavior and higher levels of light PA or MVPA could suggest a physiological mechanism to explain the healthier profile within obesity for MHO women.

Other PA variables derived from accelerometry, such as TAC and steps, although higher in MHO women, did not reach statistical significance in the current study. Interestingly, mean TAC values for MHO and MUO represent different U.S. population percentiles: MHO total activity count means represent the 50^th^ percentile, while MUO total activity count means represent the 25^th^ percentile [34]. In addition, MHO women had approximately 20 more minutes of MVPA per day, and over 2000 more steps per day, compared to MUO women. These differences may be clinically meaningful despite their lack of statistical significance. Post-hoc power calculations show that a minimum sample size of 124 was needed to show significant differences in mean values between MHO and MUO for TAC, MVPA and steps.

It is important to note that the step counts are measured by an Actigraph accelerometer in the current study. Actigraphs are known to produce higher step counts compared to pedometers that use a spring lever or pizo-electric mechanism during free-living conditions [35], due to differences in measurement and sensitivity of detecting steps. The mean steps in both MUO and MHO groups exceeded 10,000 per day; however step thresholds for health are based on spring lever pedometers, thus the current results cannot be compared to those step guidelines. Despite the differences in measurement between pedometers and the Actigraph accelerometer, the focus here is on the differences between MHO and MUO regardless of the device used.

Previous studies have utilized television viewing time as a proxy measure to compare sedentary behavior among MHO and MUO individuals and have found no significant differences [7, 13]. Although we expanded the measure of screen time to include both TV viewing and computer time, our null findings are consistent with previous reports. Interestingly, MHO women self-reported approximately 2 hours less total sitting time compared to MUO women and this difference approached significance (p = 0.06).

Higher vegetable intake has been shown to have beneficial effects on cardiovascular disease mortality [36]. MHO women reported higher vegetable and overall fiber consumption (fiber, soluble fiber, fiber from beans, and fiber from fruits and vegetables) than MUO women. Previous research has not found any differences in vegetables or total fiber intake between MHO and MUO [7, 10], but previous studies were not done in young African American and Caucasian women living in the U.S., which may explain differences in findings. We have previously reported higher whole grains, whole fruit, and meat/bean consumption in MHO young women, which typically contains higher fiber foods, however, fiber amounts were not directly investigated in the previous study [14]. Higher fiber intake has been associated with positive cardiometabolic risk factors and lower risk of obesity, cardiovascular disease and diabetes [37].

Total dairy intake was lower in our sample of MHO than MUO women. This finding is in contrast to those who reported no differences in dairy intake in other adult cohorts [7, 10, 14]. The difference in dairy intake in the current study was limited to fluid milk, and did not include cheese or yogurt. Possible explanations could be due to our 60% African American sample which are known to have lower intake of milk products due to perceived or actual lactose sensitivity [38]. We also found lower intakes of saturated fat, *trans* fat and monounsaturated fat levels in MHO. The finding for saturated fat is different than that reported in another cohort of young women [14] and middle-older aged adults [7]. The higher intakes of both vegetables and lower intake of dairy intake in the MHO women likely accounted for the lower saturated fat intakes.

There are several notable strengths and limitations to the current study. Dietary data assessment, despite quality data collection methodology and analysis, is subject to recall bias. Our sample was drawn from the Boston metropolitan and suburban communities, possibly limiting generalizability to other cities and communities. The current study is a cross-sectional design and cause and effect of the key lifestyle behaviors on cardiometabolic risk within obesity are not able to be determined. Due to our small relatively sample size (n = 54), our findings should be interpreted as preliminary; future studies are needed to replicate our findings with larger samples that include men and women of diverse race/ethnic groups. Despite these limitations, our rigorous objective monitoring for both PA and sedentary behavior using accelerometry allowed assessment of various intensities of PA and overall PA levels which has not previously been investigated in MHO research. We were able to include young African American and Caucasian women. African American young women, in particular, are more likely to be MHO [6], but are also more susceptible to developing hypertension, CVD and diabetes [39, 40], making this group a high priority for better understanding lifestyle behaviors associated with the MHO profile.

Our research shows differences in key lifestyle behaviors between young women who were classified as MHO versus MUO. National estimates suggest that adults spend approximately 55% of their waking hours in sedentary behavior [18]. Reducing or displacing time in sedentary behavior with time in PA, regardless of intensity, could be a strategy to improve health even within the setting of obesity. This cross sectional analysis could suggest possible intervention strategies which may improve cardiometabolic risk in obesity without weight loss (ie., transitioning from the MUO to the MHO phenotype) including: 1) substituting light PA for sedentary behavior and 2) dietary intake with higher vegetables, fiber and decreasing saturated fat. Current guidelines for obesity treatment emphasize weight loss utilizing multiple lifestyle behaviors and strategies [41]; however, weight loss is difficult to achieve and maintain [42]. If future studies are able to show that changes in key lifestyle behaviors such as sedentary behavior, PA and diet can be made to improve health within obesity, without weight loss, this could ultimately lead to the need for updating and stratifying obesity treatment guidelines to address different health risks within obesity.

## Supporting Information

S1 Data Project Health Minimal Data Set. (XLSX)

## References

[1] Karelis AD, St-Pierre DH, Conus F, Rabasa-Lhoret R, Poehlman ET. Metabolic and body composition factors in subgroups of obesity: what do we know? J Clin Endocrinol Metab. 2004;89(6):2569–75. 15181025 10.1210/jc.2004-0165

[2] Brochu M, Tchernof A, Dionne IJ, Sites CK, Eltabbakh GH, Sims EA, et al What are the physical characteristics associated with a normal metabolic profile despite a high level of obesity in postmenopausal women? J Clin Endocrinol Metab. 2001;86(3):1020–5. 11238480 10.1210/jcem.86.3.7365

[3] Ogorodnikova AD, Kim M, McGinn AP, Muntner P, Khan U, Wildman RP. Incident cardiovascular disease events in metabolically benign obese individuals. Obesity (Silver Spring). 2012;20(3):651–9. 21799477 10.1038/oby.2011.243 PMC3494999

[4] van der ADL, Nooyens AC, van Duijnhoven FJ, Verschuren MM, Boer JM. All-cause mortality risk of metabolically healthy abdominal obese individuals: the EPIC-MORGEN study. Obesity (Silver Spring). 2014;22(2):557–64. 23595997 10.1002/oby.20480

[5] Velho S, Paccaud F, Waeber G, Vollenweider P, Marques-Vidal P. Metabolically healthy obesity: different prevalences using different criteria. Eur J Clin Nutr. 2010;64(10):1043–51. 10.1038/ejcn.2010.114 20628408

[6] Wildman RP, Muntner P, Reynolds K, McGinn AP, Rajpathak S, Wylie-Rosett J, et al The obese without cardiometabolic risk factor clustering and the normal weight with cardiometabolic risk factor clustering: prevalence and correlates of 2 phenotypes among the US population (NHANES 1999–2004). Arch Intern Med. 2008;168(15):1617–24. 10.1001/archinte.168.15.1617 18695075

[7] Hankinson AL, Daviglus ML, Van Horn L, Chan Q, Brown I, Holmes E, et al Diet composition and activity level of at risk and metabolically healthy obese American adults. Obesity (Silver Spring). 2013;21(3):637–43. 23592673 10.1002/oby.20257 PMC3416914

[8] Lee K. Metabolically obese but normal weight (MONW) and metabolically healthy but obese (MHO) phenotypes in Koreans: characteristics and health behaviors. Asia Pac J Clin Nutr. 2009;18(2):280–4. 19713189

[9] Messier V, Karelis AD, Prud'homme D, Primeau V, Brochu M, Rabasa-Lhoret R. Identifying metabolically healthy but obese individuals in sedentary postmenopausal women. Obesity (Silver Spring). 2010;18(5):911–7. 19851302 10.1038/oby.2009.364

[10] Phillips CM, Dillon C, Harrington JM, McCarthy VJ, Kearney PM, Fitzgerald AP, et al Defining metabolically healthy obesity: role of dietary and lifestyle factors. PLoS One. 2013;8(10):e76188 10.1371/journal.pone.0076188 24146838 PMC3798285

[11] Artero EG, Lee DC, Lavie CJ, Espana-Romero V, Sui X, Church TS, et al Effects of muscular strength on cardiovascular risk factors and prognosis. J Cardiopulm Rehabil Prev. 2012;32(6):351–8. 10.1097/HCR.0b013e3182642688 22885613 PMC3496010

[12] Sarvottam K, Magan D, Yadav RK, Mehta N, Mahapatra SC. Adiponectin, interleukin-6, and cardiovascular disease risk factors are modified by a short-term yoga-based lifestyle intervention in overweight and obese men. J Altern Complement Med. 2013;19(5):397–402. 10.1089/acm.2012.0086 23210469

[13] Bell JA, Kivimaki M, Batty GD, Hamer M. Metabolically healthy obesity: what is the role of sedentary behaviour? Prev Med. 2014;62:35–7. 10.1016/j.ypmed.2014.01.028 24513171 PMC3995089

[14] Camhi SM, Whitney Evans E, Hayman LL, Lichtenstein AH, Must A. Healthy eating index and metabolically healthy obesity in U.S. adolescents and adults. Prev Med. 2015;77:23–7. 10.1016/j.ypmed.2015.04.023 25937589

[15] Li S, Chen W, Srinivasan SR, Xu J, Berenson GS. Relation of childhood obesity/cardiometabolic phenotypes to adult cardiometabolic profile: the Bogalusa Heart Study. Am J Epidemiol. 2012;176 Suppl 7:S142–9. 10.1093/aje/kws236 23035138 PMC3530363

[16] Cornier MA, Despres JP, Davis N, Grossniklaus DA, Klein S, Lamarche B, et al Assessing adiposity: a scientific statement from the american heart association. Circulation. 2011;124(18):1996–2019. 10.1161/CIR.0b013e318233bc6a 21947291

[17] Matthews DR, Hosker JP, Rudenski AS, Naylor BA, Treacher DF, Turner RC. Homeostasis model assessment: insulin resistance and beta-cell function from fasting plasma glucose and insulin concentrations in man. Diabetologia. 1985;28(7):412–9. 3899825 10.1007/BF00280883

[18] Matthews CE, Chen KY, Freedson PS, Buchowski MS, Beech BM, Pate RR, et al Amount of time spent in sedentary behaviors in the United States, 2003–2004. Am J Epidemiol. 2008;167(7):875–81. 10.1093/aje/kwm390 18303006 PMC3527832

[19] Matthews CE. Calibration of Accelerometer Output for Adults. Med Sci Sports Exerc. 2005;37(11):s512–s22. 16294114 10.1249/01.mss.0000185659.11982.3d

[20] Crouter SE, DellaValle DM, Haas JD, Frongillo EA, Bassett DR. Validity of ActiGraph 2-regression model, Matthews cut-points, and NHANES cut-points for assessing free-living physical activity. J Phys Act Health. 2013;10(4):504–14. 22975460 10.1123/jpah.10.4.504 PMC4199088

[21] Tudor-Locke C, Johnson WD, Katzmarzyk PT. Accelerometer-determined steps per day in US adults. Med Sci Sports Exerc. 2009;41(7):1384–91. 10.1249/MSS.0b013e318199885c 19516163

[22] Crouter SE, Kuffel E, Haas JD, Frongillo EA, Bassett DR Jr. Refined two-regression model for the ActiGraph accelerometer. Med Sci Sports Exerc. 2010;42(5):1029–37. 10.1249/MSS.0b013e3181c37458 20400882 PMC2891855

[23] Troiano RP, Berrigan D, Dodd KW, Masse LC, Tilert T, McDowell M. Physical activity in the United States measured by accelerometer. Med Sci Sports Exerc. 2008;40(1):181–8. 18091006 10.1249/mss.0b013e31815a51b3

[24] Blair SN, Haskell WL, Ho P, Paffenbarger RS Jr., Vranizan KM, Farquhar JW, et al Assessment of habitual physical activity by a seven-day recall in a community survey and controlled experiments. AmJEpidemiol. 1985;122(5):794–804. 10.1093/oxfordjournals.aje.a114163 3876763

[25] Sallis JF, Haskell WL, Fortmann SP, Vranizan KM, Taylor CB, Solomon DS. Predictors of adoption and maintenance of physical activity in a community sample. Prev Med. 1986;15(4):331–41. 3763558 10.1016/0091-7435(86)90001-0

[26] Washburn RA, Jacobsen DJ, Sonko BJ, Hill JO, Donnelly JE. The validity of the Stanford Seven-Day Physical Activity Recall in young adults. Med Sci Sports Exerc. 2003;35(8):1374–80. 12900693 10.1249/01.MSS.0000079081.08476.EA

[27] NHANES Questionnaires,Datasets, and Related Documentation Hyattsville, MD: National Center for Health Statistics; 2012 [cited 2015 January 12]. Available: http://www.cdc.gov/nchs/nhanes/nhanes_questionnaires.htm.

[28] Craig CL, Marshall AL, Sjostrom M, Bauman AE, Booth ML, Ainsworth BE, et al International physical activity questionnaire: 12-country reliability and validity. Med Sci Sports Exerc. 2003;35(8):1381–95. 12900694 10.1249/01.MSS.0000078924.61453.FB

[29] Block G, Woods M, Potosky A, Clifford C. Validation of a self-administered diet history questionnaire using multiple diet records. Journal of clinical epidemiology. 1990;43(12):1327–35. 2254769 10.1016/0895-4356(90)90099-b

[30] Healy GN, Dunstan DW, Salmon J, Cerin E, Shaw JE, Zimmet PZ, et al Objectively measured light-intensity physical activity is independently associated with 2-h plasma glucose. Diabetes Care. 2007;30(6):1384–9. 17473059 10.2337/dc07-0114

[31] Duvivier BM, Schaper NC, Bremers MA, van Crombrugge G, Menheere PP, Kars M, et al Minimal intensity physical activity (standing and walking) of longer duration improves insulin action and plasma lipids more than shorter periods of moderate to vigorous exercise (cycling) in sedentary subjects when energy expenditure is comparable. PLoS One. 2013;8(2):e55542 10.1371/journal.pone.0055542 23418444 PMC3572053

[32] Buman MP, Hekler EB, Haskell WL, Pruitt L, Conway TL, Cain KL, et al Objective light-intensity physical activity associations with rated health in older adults. Am J Epidemiol. 2010;172(10):1155–65. 10.1093/aje/kwq249 20843864 PMC3004766

[33] Hamer M, Stamatakis E, Steptoe A. Effects of substituting sedentary time with physical activity on metabolic risk. Med Sci Sports Exerc. 2014;46(10):1946–50. 10.1249/MSS.0000000000000317 24674977 PMC4186723

[34] Wolff DL, Fitzhugh EC, Bassett DR, Churilla JR. Waist-Worn Actigraphy: Population-Referenced Percentiles for Total Activity Counts in U.S. Adults. J Phys Act Health. 2014;6 4. [Epub ahead of print]. 10.1123/jpah.2013-0464 24905055

[35] Tudor-Locke C, Ainsworth BE, Thompson RW, Matthews CE. Comparison of pedometer and accelerometer measures of free-living physical activity. Med Sci Sports Exerc. 2002;34(12):2045–51. 12471314 10.1097/00005768-200212000-00027

[36] Leenders M, Boshuizen HC, Ferrari P, Siersema PD, Overvad K, Tjonneland A, et al Fruit and vegetable intake and cause-specific mortality in the EPIC study. Eur J Epidemiol. 2014;29(9):639–52. 10.1007/s10654-014-9945-9 25154553

[37] Anderson JW, Baird P, Davis RH Jr., Ferreri S, Knudtson M, Koraym A, et al Health benefits of dietary fiber. Nutr Rev. 2009;67(4):188–205. 10.1111/j.1753-4887.2009.00189.x 19335713

[38] Bailey RK, Fileti CP, Keith J, Tropez-Sims S, Price W, Allison-Ottey SD. Lactose intolerance and health disparities among African Americans and Hispanic Americans: an updated consensus statement. J Natl Med Assoc. 2013;105(2):112–27. 24079212 10.1016/s0027-9684(15)30113-9

[39] Kumanyika SK. Special issues regarding obesity in minority populations. Ann Intern Med. 1993;119(7 Pt 2):650–4. 8363191 10.7326/0003-4819-119-7_part_2-199310011-00005

[40] Kumanyika SK. Obesity in minority populations: an epidemiologic assessment. Obes Res. 1994;2(2):166–82. 16353618 10.1002/j.1550-8528.1994.tb00644.x

[41] Executive summary of the clinical guidelines on the identification, evaluation, and treatment of overweight and obesity in adults. ArchInternMed. 1998;158(17):1855–67. 10.1001/archinte.158.17.1855 9759681

[42] Sarwer DB, von Sydow Green A, Vetter ML, Wadden TA. Behavior therapy for obesity: where are we now? Current opinion in endocrinology, diabetes, and obesity. 2009;16(5):347–52. 10.1097/MED.0b013e32832f5a79 19623061

